# Confirmatory Efficacy and Safety Trial of Magnetic Seizure Therapy for Depression (CREST-MST): study protocol for a randomized non-inferiority trial of magnetic seizure therapy versus electroconvulsive therapy

**DOI:** 10.1186/s13063-021-05730-7

**Published:** 2021-11-08

**Authors:** Zafiris J. Daskalakis, Carol Tamminga, Alanah Throop, Lucy Palmer, Julia Dimitrova, Faranak Farzan, Kevin E. Thorpe, Shawn M. McClintock, Daniel M. Blumberger

**Affiliations:** 1 Department of Psychiatry, University of California, San Diego, La Jolla, CA USA; 2 Department of Psychiatry, University of Texas Southwestern Medical Center, Dallas, TX USA; 3 Department of Psychology, University at Buffalo, The State University of New York | SUNY Buffalo, Buffalo, USA; 4 School of Mechatronic Systems Engineering, Simon Fraser University, Surrey, British Columbia Canada; 5 Applied Health Research Centre, Li Ka Shing Knowledge Institute of St. Michael’s, Toronto, Ontario Canada; 6 Dalla Lana School of Public Health, University of Toronto, Toronto, Ontario Canada; 7 Institute of Medical Science and Department of Psychiatry, University of Toronto, Toronto, Ontario Canada; 8 Temerty Centre for Therapeutic Brain Intervention, Centre for Addiction and Mental Health, Toronto, Ontario Canada

**Keywords:** Depression, Major depressive disorder, Treatment-resistant depression, Magnetic seizure therapy, Electroconvulsive therapy, Brain stimulation, Randomized controlled trial

## Abstract

**Background:**

Electroconvulsive therapy (ECT) is well-established and effective for treatment-resistant depression (TRD), but in Canada and the USA, less than 1% of patients with TRD receive ECT mainly due to its cognitive adverse effects (i.e. amnesia). Thus, new treatment alternatives for TRD are urgently needed. One such treatment is magnetic seizure therapy (MST). ECT involves applying a train of high-frequency electrical stimuli to induce a seizure, whereas MST involves applying a train of high-frequency magnetic stimuli to induce a seizure.

**Methods:**

In this manuscript, we introduce our international, two-site, double-blinded, randomized, non-inferiority clinical trial to develop MST as an effective and safe treatment for TRD. This trial will compare the efficacy of MST to right unilateral ultra-brief pulse width electroconvulsive therapy (RUL-UB-ECT) with a combined primary endpoint of remission of depression and superior cognitive adverse effects in 260 patients with TRD. Amelioration of suicidal ideation will be assessed as a secondary endpoint. Inpatients or outpatients, over 18 years of age with a MINI International Neuropsychiatric Interview (MINI) diagnosis of non-psychotic major depressive disorder (MDD) can be enrolled in the study provided that they meet illness severity and full eligibility criteria. Participants are randomized to receive MST or RUL-UB ECT, 2-3 days per week over seven weeks, or a maximum of 21 treatments. The study will involve before-, during-, and after-treatment assessments of depression severity, suicidal ideation, subjective side-effects, and cognitive performance consistent with an intent-to-treat study design approach.

**Discussion:**

Positive results from this trial could have an immediate and tremendous impact for patients with TRD. If MST demonstrates comparable antidepressant treatment efficacy to ECT, but with greater cognitive safety, it could rapidly be adopted into clinical practice. Indeed, given that the administration of MST is nearly identical to ECT, the majority of ECT facilities in North America could readily adopt MST. Furthermore, the potential for cognitive safety could lead to improved treatment acceptability. Healthcare providers, patients and care partners, and policymakers would therefore demand this form of convulsive therapy.

**Trial status:**

Enrollment for this study began on June 26, 2018, and is estimated to complete recruitment by July 2024. At the time of submission, we have enrolled and randomized 117 participants.

**Trial registration:**

ClinicalTrials.gov NCT03191058, Registered on June 19, 2017.

Primary sponsor:

Daniel Blumberger (DMB), Principal Investigator

Daniel.Blumberger@camh.ca, 416-535-8501 x 33662

Contact for public queries: DMB, Daniel.Blumberger@camh.ca

Contact for scientific queries: ZJD, Zdaskalakis@health.ucsd.edu

**Supplementary Information:**

The online version contains supplementary material available at 10.1186/s13063-021-05730-7.

## Background

In this National Institute of Mental Health (NIMH) funded study, we aim to assess the efficacy and tolerability of magnetic seizure therapy (MST) as an alternative to electroconvulsive therapy (ECT) for major depressive disorder (MDD). Even with multiple antidepressant medication trials, 30 - 40% of patients will experience a pharmacologically resistant form of MDD (i.e. treatment-resistant depression (TRD)) [1]. In the USA, the total economic burden of MDD as of 2010 was estimated to be $210.5 billion per year, representing a 21.5% increase from $173.2 billion per year in 2005 [2]. The ineffectiveness of current antidepressant treatments coupled with the economic burden associated with depression provides a strong rationale for the development of new and safe antidepressant therapeutic interventions that can provide greater response and remission rates.

While ECT is a well-established and highly effective treatment for depression with remission rates ranging from 60% to 80 %[3], less than 1% of patients with TRD receive ECT in Canada and the USA [4, 5]. One of the major reasons that providers, patients, and their families refuse to consider ECT, even when confronted with disabling and life-threatening depression, is concern about the ECT-induced cognitive side effects (e.g. amnesia). Regardless of treatment delivery, ECT results in significant cognitive impairment including post-ictal disorientation, anterograde and retrograde amnesia, and executive dysfunction [6]. Disorientation immediately after ECT can last up to 60 min, regardless of electrode placement [7], which lengthens and can complicate recovery time after the procedure and causes patient distress and burden [8]. Anterograde amnesia, the inability to learn and retain new information, can appear at the first ECT treatment [9, 10] and persist for up to several months [11–14]. Retrograde amnesia, the inability to recall past personal and impersonal memories, remains one of the most distressing ECT cognitive adverse effects [15]. Retrograde amnesia has been found to be more severe with bitemporal than right unilateral (RUL) ECT, with high compared to low dosages, and with sine wave and brief pulse width relative to ultrabrief pulse width[6]. As recently highlighted by the US Food and Drug Administration (FDA), the extent of anterograde and retrograde amnesia, and the degree to which memory remains impaired post-ECT is a significant problem for patients and their families [16]. Indeed, numerous patients and their families refuse ECT because of these adverse cognitive effects.

Several theories have been proposed to account for the ECT-induced adverse effects on memory. The most supported theory is that when an electrical seizure is initiated, the skull shunts the electrical current away from the stimulation site. This electrical current is propagated throughout the brain by the corticospinal fluid and ensures the electrical stimulus is non-focal [17, 18]. This diffuse electrical discharge spreads to deep brain regions, including medial temporal lobe structures (e.g. hippocampus), causing disruption of synaptic plasticity and long-term potentiation [19], which are neural substrates of learning and memory formation [20–23]. The development of a new treatment approach that can mitigate these adverse cognitive effects, while maintaining the robust efficacy of ECT, is significantly needed. MST offers a viable, and arguably favourable, alternative to ECT with comparable antidepressant response/remission rates and a more favourable cognitive safety profile [24–26].

MST is an alternative form of convulsive therapy, that like ECT, involves the induction of a seizure to achieve a therapeutic effect [18]. However, the induction of a seizure occurs through high-frequency, repetitive transcranial magnetic stimulation (rTMS) rather than high-frequency, repetitive transcranial electrical stimulation. With magnetic stimulation, a rapid, high intensity, time-varying magnetic field is able to pass into the brain without resistance thereby limiting seizure spread. The induced magnetic field can be focally targeted to stimulate specific neurons based on geometry of the stimulating magnetic coil [27, 28]. Additionally, the magnetic field is not impeded nor shunted by non-conducting material (i.e. skull) and, therefore, results in focal brain activation [29, 30]. Specifically, compared to RUL-UB ECT, the electric field induced by MST is 5–10 times more focal [30, 31].

In an early study, White et al. compared 20 patients with severe depression openly allocated to receive ECT or MST. In the MST relative to the ECT group, time to orientation, a measure of post-ictal disorientation, was significantly shorter (4 vs. 18 min, *p*< 0.01). The Hamilton Rating Scale for Depression-24 item (HRSD-24) total score improved from 32 to 14 in the MST group, while in the ECT group, the total score improved from 30 to 6 [32]. In another study, 20 patients with TRD were allocated to receive either RUL-ECT or MST. Comparable antidepressant response and no cognitive side effects were observed in the two groups [26]. Of note in that latter study, RUL-ECT was administered at 3 times the seizure threshold, which reduced the cognitive side effects and could have minimized the ECT-induced antidepressant benefits. A subsequent MST study conducted by the same group collated the data from the former study with 16 additional adults who received MST. Remission was achieved by 46% of the patients and no cognitive side effects were observed. Fluorodeoxyglucose positron emission tomography (FDG-PET) scans (*N*=12) showed increased bilateral metabolic activity in the frontal cortex and decreased activity in the left striatum [33]. Based on theoretical calculations, Lee et al. found the electrical field to be 3–11 times stronger and the stimulated brain volume much larger (47–100% vs 21%) with ECT compared to MST. The improved focality and lower intensity of MST were suggested as a possible explanation for its favourable cognitive safety profile [31]. In a within-subject, non-human primate study, McClintock et al. compared the effects of electroconvulsive seizure (ECS), magnetic seizure (MS), and anaesthesia alone on a measure of spatial working memory. ECS resulted in a lower correlation between predicted and actual response patterns in 2 of 3 subjects, which suggested impaired planning ability. In all 3 subjects, reaction time was significantly longer in ECS relative to MS and sham [34]. Relative to ECS, this preclinical study substantiated the cognitive safety and superiority of MST.

In a recent published study from our group, we found support for favourable antidepressant effects of MST with a larger sample size in an open-label study design [35]. We evaluated the antidepressant and neurocognitive effects of MST applied over the prefrontal cortex at low (25 Hz), medium (50 or 60 Hz), and high (100 Hz) frequencies. The primary analysis examined 86 adult patients with MDD who completed an adequate treatment course (i.e. 8 MST treatments or more) and 47 patients who completed the study per protocol either having achieved remission (i.e. HRSD-24 total score ≤ 10 and a comparative reduction of > 60% on two consecutive assessments) or received a maximum of 24 treatment sessions [35]. This study employed before-, during-, and after-treatment clinical and cognitive monitoring assessments. Response (i.e. 50% reduction on the HRSD-24) and remission were assessed weekly throughout the trial. High (100 Hz) frequency MST produced the largest response and remission rates for both adequate trial completers (41.7% and 33.3%, respectively) and protocol completers (60% and 53.3%, respectively). Furthermore, we found that MST has negligible effects on neurocognitive function. Overall, our findings suggest that the implementation of frontal stimulation at high frequency produces effective depression response and remission rates that may be comparable to ECT, but with greater cognitive safety.

In this manuscript, we present our international, NIMH-funded, double-blinded, randomized, non-inferiority study protocol that evaluates the efficacy, tolerability, and cognitive adverse effects of MST compared to RUL-UB ECT (research protocol version 12.0; 21 Oct. 2020). Several important areas of innovation are included in this study. First, is the use of a relatively new non-invasive neuromodulation therapy, MST. Second, is the non-inferiority clinical trial design to compare the efficacy of MST with ECT in adult patients with TRD. Finally, suicidal ideation—a symptom domain in TRD that has tremendous public health impact—is evaluated as a secondary outcome variable of interest. Other innovations include state-of-the-art combined TMS with electroencephalography (EEG) and analytic methods used to identify a neurophysiological GABAergic signal, and the use of quantitative EEG to probe mechanisms of ECT-induced cognitive impairment. In the current manuscript, we outline the protocol for the clinical and cognitive outcomes of the trial. An accompanying second manuscript details the neurophysiology protocol [36].

## Methods

### Study design and setting

**Fig. 1:**
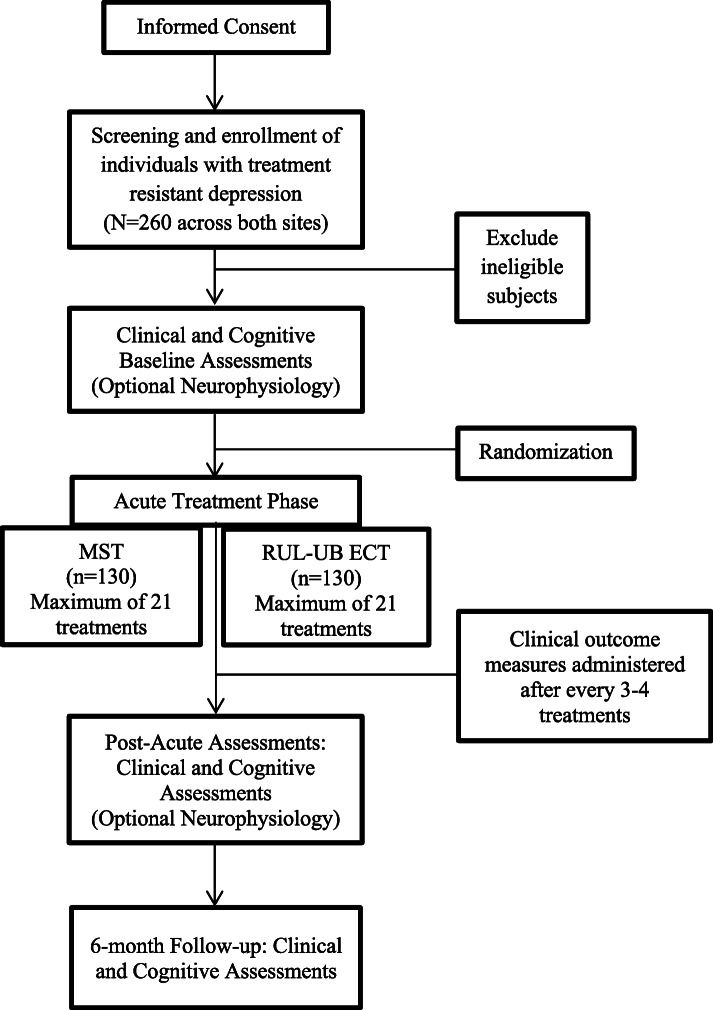
Study design

The study involves a double-blinded, randomized, non-inferiority clinical trial with two treatment arms (see Fig. 1: Study design) conducted at two leading academic institutions in North America: the Temerty Centre for Therapeutic Brain Intervention at the Centre for Addiction and Mental Health (CAMH) in Toronto, Ontario, Canada, and University of Texas Southwestern (UT Southwestern) Medical Center in Dallas, Texas, USA. A total of 260 adult patients with MDD will be randomized to receive MST or RUL-UB-ECT.

Treatment is administered 2 to 3 days per week. Depression symptoms and severity are assessed with the HRSD-24 [37] and suicidality is assessed with the Beck Scale for Suicidal Ideation (SSI) [38]. Remission is defined as HRSD-24 total score ≤ 10 and a ≥ 60% decrease in total score from baseline on two consecutive ratings. Remission of suicidal ideation is defined as a score of 0 on the SSI. Therefore, there is no specific minimum number of treatments that patients must receive to be classified as remitters. However, patients who do not meet remission criteria after 21 treatment sessions are considered non-remitters and cease treatment sessions. This maximum treatment number was chosen to allow for the possibility that MST may require more treatment sessions to achieve remission, similar to RUL-UB ECT [39–41]. The study blind will not be broken to participants except in the case of physician safety concerns.

The clinical trial study was approved by the research ethics boards of CAMH (Reference Number 033-2017) and UT Southwestern (Reference Number STU 032017-022). The trial was issued an Investigational Device Exemption (IDE; Reference Number G170127) by the U.S. FDA and an Investigational Testing Authorization (ITA; Reference Number 270547) by Health Canada to assess the safety and efficacy of the MST MagPro XP with Cool Twin Coil (MagVenture A/S, Farum, Denmark) within this clinical trial. The study is registered with ClinicalTrials.gov under the identifier NCT03191058 and results will be reported in a manner consistent with the international Consolidated Standards of Reporting Trials (CONSORT) guidelines. The research ethics boards of both study site institutions involved in the study are notified if any changes are made to the study protocol. The trial registration is also updated as appropriate. This protocol is in accordance with the Standard Protocol Items: Recommendations for Interventional Trials (SPIRIT) [42] guidelines (see Additional file 1).

### Recruitment and retention

To ensure we meet our recruitment goals, both sites have implemented new innovations including treatment care pathways that ensure many patients with TRD are offered brain stimulation treatments should they be unresponsive to initial pharmacological approaches. Brain stimulation psychiatrists are informed about clinical research and trained to screen all new referrals for potential recruitment. The CREST-MST study has recruitment milestones for overall recruitment as well as racial and ethnic minority recruitment to ensure a representative sample is obtained. Retention strategies have been implemented to mitigate patient discontinuation throughout the study including constant communication between study staff and patient, weekly check-ins with the study psychiatrist, and an intent to treat (ITT) approach whereby patients continue to be followed and offered alternative treatment even if discontinued from the trial.

### Eligibility criteria

Patients are included in the study if they (1) are inpatients or outpatients; (2) are voluntary and competent to consent to treatment and research procedures according to an ECT/MST attending psychiatrist; (3) have a MINI International Neuropsychiatric Interview diagnosis of non-psychotic MDD; (4) are 18 years of age or older; (5) have a baseline HRSD-24 score ≥ 21; (6) are considered to be appropriate to receive convulsive therapy; (7) are agreeable to keeping their current antidepressant treatment constant during the intervention; (8) are likely able to adhere to the intervention schedule; (9) meet the MST safety criteria [43]; and (10) if a woman of child-bearing potential: is willing to provide a negative pregnancy test and agrees not to become pregnant during trial participation. Patients are excluded from the study if they (1) have a history of a MINI diagnosis of substance dependence or abuse within the past three months; (2) have a concomitant major unstable medical illness; (3) are pregnant or intend to get pregnant during the study; (4) have a MINI diagnosis of any primary psychotic disorder; (5) have a MINI diagnosis of obsessive compulsive disorder, or post-traumatic stress disorder deemed to be primary and causing more functional impairment than the depressive disorder; (6) have probable dementia; (7) have any significant neurological disorder or condition likely to be associated with increased intracranial pressure or a space occupying brain lesion; (8) present with a medical condition, a medication, or a laboratory abnormality that could cause a major depressive episode or significant cognitive impairment in the opinion of the investigator; (9) have an intracranial implant or any other metal object within or near the head, excluding the mouth, that cannot be safely removed; (10) require a benzodiazepine with a dose > lorazepam 2 mg/day or equivalent or any anticonvulsant; (11) are unable to communicate in English fluently enough to complete the neuropsychological tests; and (12) have a non-correctable clinically significant sensory impairment. These eligibility criteria are congruent with the criteria that have been used in the major ECT trials conducted during the past decade [6, 44, 45].

### Informed consent procedures

At both study sites, general physicians or psychiatrists refer patients for an initial consultation with a brain stimulation psychiatrist to assess suitability for convulsive therapy and are then referred to qualified research personnel. The qualified research personnel will then explain the trial in terms suited to the patient’s comprehension of the purposes, procedures, and potential risks of the study, and of their rights as research participants. If participants would like to proceed an eligibility screening assessment is scheduled. Patients are provided with a consent form (Additional file 2) describing in detail the study intervention, study procedures, and risks, and all questions are answered. Written documentation of informed consent is required prior to initiating the screening visit to assess for eligibility. The consent form includes an additional signature line for the collection of neurophysiological biomarkers discussed in detail in an accompanying manuscript [36]. Once consent is obtained according to Institutional Review Board and Good Clinical Practice (GCP)/Tri-Council guidelines, the research personnel confirm inclusion/exclusion criteria is met with the site Principal Investigator (PI) before proceeding with baseline testing. Patients are informed that they can withdraw participation at any point during the study. Further, patients are informed of any approved protocol changes at their next study visit and re-consented if applicable. The rights and welfare of the participants are protected by emphasizing to them that the quality of their medical care will not be adversely affected if they decline to participate in this study.

### Randomization and blinding

Upon the completion of informed consent and the collection of baseline data, consenting and eligible participants are randomized to receive either MST or RUL-UB ECT using a permuted block method with a random number generator using blocks of varying sizes. Study personnel are blinded to the randomization block sizes. The Applied Health Research Centre (AHRC) at St. Michael’s Hospital (SMH) centrally manages the randomization of participants. The random permuted blocks and central randomization ensure allocation concealment. Although the treatment team administering MST or ECT cannot be blind, all patients remain blind to their treatment assignment during the course of the entire study. Similarly, the independent raters administering the efficacy and tolerability outcome assessments, as well as the neuropsychological raters, remain blind to treatment assignment during the entire study. Breaking the blind for a single patient will only be considered when knowledge of the treatment assignment is deemed essential by the physician for patient care. To assess the integrity of blinding procedures, participants and raters are asked to complete a conventional guess form asking them whether they believe participants received MST or RUL-UB ECT after the participant has received their first treatment.

### Interventions

**Fig. 2:**
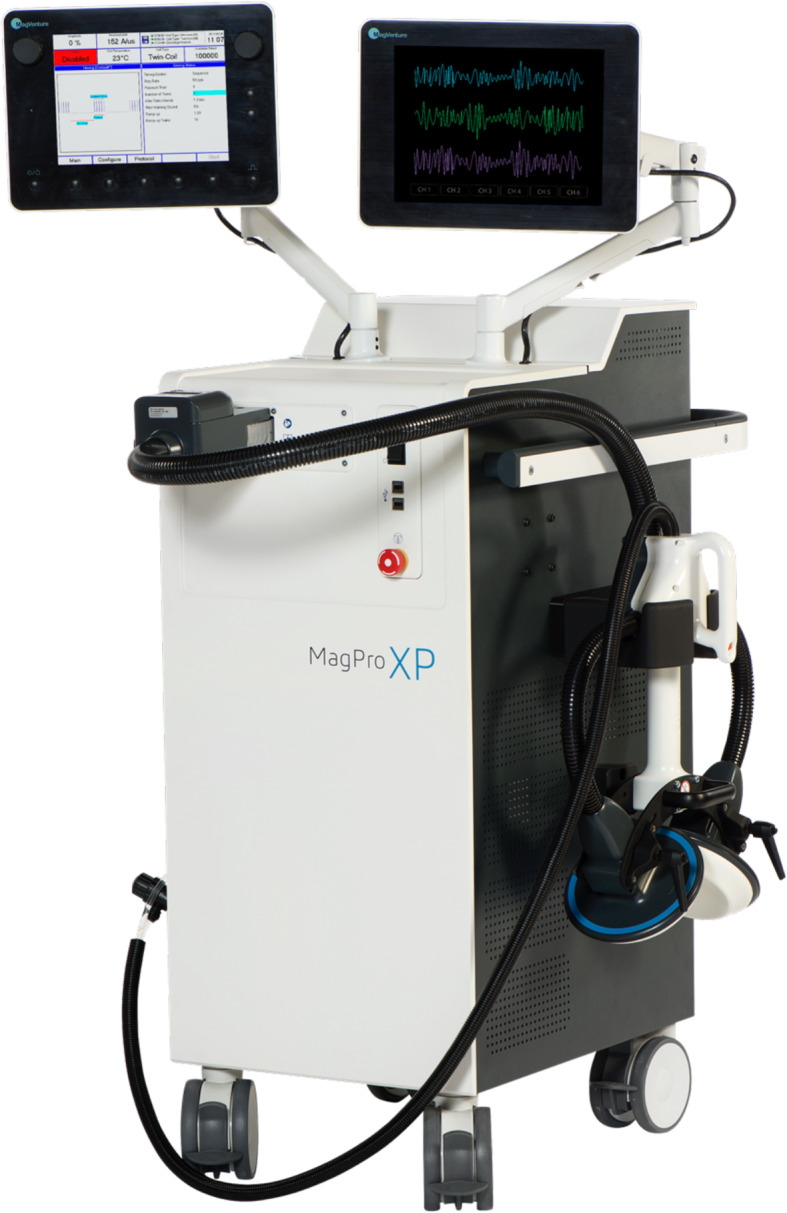
MagPro XP with Cool Twin Coil (MagVenture A/S, Farum, Denmark)

A total of up to 21 treatments are administered to participants, two to three times a week. At all sites, treatment with either MST or RUL-UB ECT is provided by trained study psychiatrists and follows standard protocols. Anesthesiologists experienced in convulsive therapy administer general anaesthesia using methohexital or etomidate, muscle relaxation using succinylcholine, and mask ventilation with 100% oxygen. Following a standard established protocol [46], a study psychiatrist will determine the seizure threshold during the first treatment. MST treatments are administered using the MagPro XP MST with Cool Twin Coil (see Fig. 2: MagPro XP). Stimulation is delivered over the frontal cortex at the midline position. By delivering convulsive stimuli to frontal brain regions, relative to other MST studies [35, 47–49], this study has an advanced MST treatment paradigm. The MST determination of seizure threshold is done using 100% machine output applied at 100 Hz at progressively escalating train durations, commencing at 2 s and increasing by 2 s with each subsequent stimulation until an adequate seizure is produced. During subsequent MST sessions, a single stimulation is delivered using a train duration that is 4 s longer than the train duration at threshold (up to a maximum train duration of 10 s). ECT treatments are administered using the MECTA spECTrum 5000Q (MECTA Corporation, Tualatin, Oregon, USA). The ECT determination of seizure threshold and the adjustment of energy at subsequent ECT sessions is based on a standard published protocol [10]. Participants will receive RUL-UB ECT at six times the seizure threshold. This approach follows the treatment paradigms of prior ECT trials [44, 45].

During all sessions, the seizure quality will be monitored using fronto-mastoid EEG. Congruent with published criteria, seizures will be considered adequate if they result in generalized tonic-clonic activity > 15 s of motor tonic-clonic activity [45, 50, 51], including the duration of the stimulus [52]. The anaesthetic dosing and MST or ECT parameters will be reviewed and optimized in the event of inadequate seizures. If the seizure produced is inadequate, a second stimulation is administered during the same session at stimulus intensity 25% above the level that resulted in the inadequate seizure, up to a maximum output of 568.3 mC for ECT or using a train duration that is 1 s longer to a maximum of 10 s for MST. If seizure duration still remains below the motor duration cut-off, then the seizure is accepted for that particular treatment.

Medications that may be used to treat MST or ECT related side effects include, but are not limited to granisetron, ondansetron, or dimenhydrinate for nausea; ketorolac, acetaminophen, or ibuprofen for headaches or muscle pain; and esmolol or labetolol for treatment-related hypertension. Prolonged seizures (i.e. seizures longer in duration than 2 min as recorded either through EEG (spike-wave complexes) or through prolonged tonic-clonic muscular activity) will be treated with either repeat administration of the anaesthetic (i.e. methohexital) or midazolam. Midazolam will be used as judiciously as possible owing to its potential to prolong reorientation times after treatment. Medications are used at doses within labelling.

If the patient fails to achieve an equal or greater than 25% decrease on the HRSD-24 total score from baseline following treatment six, the charge is increased by approximately 50% for ECT or increased by 200 pulses for MST. This process is repeated based on the HRSD-24 total scores from both treatments 9 and 12, and parameters are adjusted accordingly. If at any point the patient is already at maximum stimulation (568.3 mC or 1000 pulses) the treatment continues with the parameters unchanged.

Participants are discontinued from the treatment if they cannot safely continue the study based on any of the following criteria: (1) experience worsening in depression severity, defined as an increase in the HRSD-24 total score from baseline of more than 30% on two consecutive assessments; (2) experience clinically significant increase in suicidal ideation with imminent intent (based on the SSI) or attempt suicide; (3) develop clinically significant hypomanic or manic symptoms; (4) emergence of catatonia; (5) withdraw consent; (6) the PI believes that for safety reasons it is in the best interest of the participant to stop participation; (7) participant engages in a serious attempt to harm others; (8) missed seizures (i.e. no induced seizure) on 2 consecutive treatment sessions despite parameter and anaesthesia optimization; and (9) non-compliant with treatment schedule. Consistent with an ITT approach, willing participants who are discontinued from treatment will not be removed from the trial entirely. They will continue to be followed and will complete post-treatment assessments to the extent possible, contingent upon patient agreement. The study PI reserves the right to fully discontinue participants from the trial if they believe it is in the best interests of the participant or study staff.

### Study schedule

The study schedule of events is described in Table 1: Study measures. Prior to screening, capacity to consent to treatment and research procedures will be assessed and documented as previously described. Prior to the acute treatment phase, baseline data is collected using clinical and cognitive outcome measures. Data is collected by trained and certified clinical and cognitive raters directly into the electronic data capture system, Medidata Rave (RAVE), or onto paper source documents where necessary. Rater administered clinical assessments are captured in RAVE while self-report questionnaires and cognitive assessments are completed on paper and transcribed into the RAVE database. Hard copy completed data collection forms are stored at each site. Clinical outcome measures are completed at baseline, after every three or four treatments immediately prior to the next treatment session, within four days of the last treatment session, and then six months post-treatment. This latter time point is part of an exploratory analysis to study the long-term clinical and cognitive outcomes post-ECT or post-MST treatment. The cognitive battery was designed to comprehensively examine cognitive dimensions that are affected by seizure therapies while minimizing the assessment burden on participants [19]. Assessments included in the cognitive battery measure performance validity, global cognitive function, estimated pre-morbid intellectual ability, attention, processing speed, verbal fluency, verbal and visual learning and memory, autobiographical memory, working memory, and executive functions (e.g. complex planning, inhibition, cognitive flexibility). Neurocognitive assessments are completed at baseline, upon participant termination of treatment, and six months post-treatment. Raters inquire about adverse events (AE) at every treatment and study visit; any event endorsed by a patient is recorded in the RAVE database. In addition to the clinical and neurocognitive measurements, neurophysiological measures for biomarkers are completed both at baseline and upon participant termination of treatment [36].

**Table 1:**
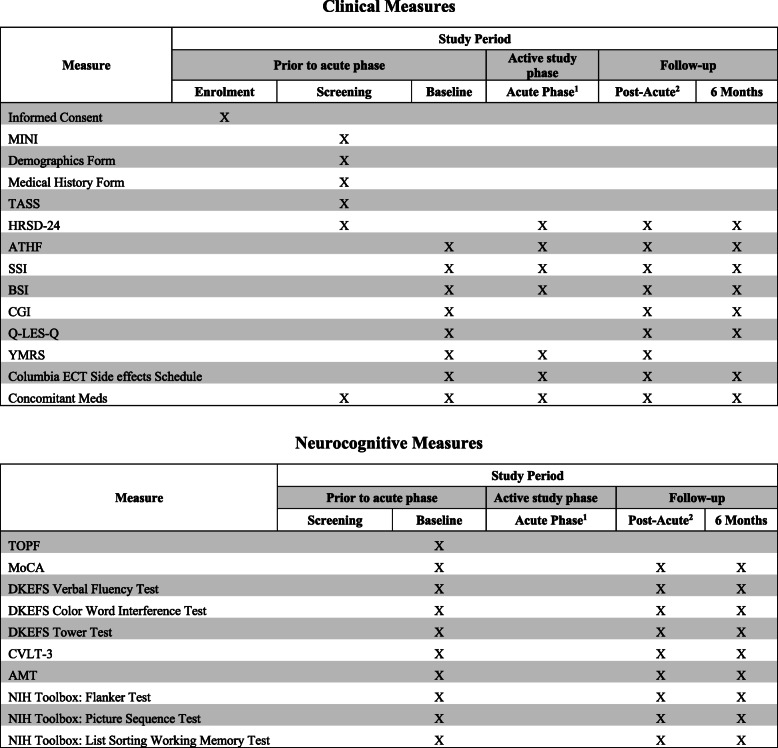
Study measures

^1^Acute treatments are delivered 2-3 times a week. Acute assessments are delivered after every 3-4 treatments

^2^≤ 4 days post-acute phase

Abbreviations:

MINI International Neuropsychiatric Interview diagnosis V6.0 (MINI), Transcranial Magnetic Stimulation Adult Safety Screen (TASS), 24-item Hamilton Depression Rating Scale (HRSD-24), Antidepressant Treatment History Form (ATHF), Scale for Suicidal Ideation (SSI), Brief Symptom Inventory (BSI), Clinical Global Impression Scale (CGI), Quality of Life Enjoyment and Satisfaction Questionnaire (Q-LES-Q), Young Mania Rating scale (YMRS), Test of Premorbid Function (TOPF), Montreal Cognitive Assessment (MoCA), Delis Kaplan Executive Function System (DKEFS), California Verbal Learning Test- Third Edition (CVLT-3), Autobiographical Memory Test (AMT), National Institutes of Health (NIH) Toolbox: Flanker Inhibitory Control and Attention Test, Picture Sequence Memory Test, List Sorting Working Memory Test

### Outcomes

The primary objective for this trial is to assess the efficacy and cognitive adverse effects of MST compared to RUL-UB ECT in patients with MDD. The primary hypotheses include (1) MST will result in remission rates that is non-inferior to that of RUL-UB ECT; and (2) MST will have a superior cognitive adverse effect and tolerability profile compared to RUL-UB ECT. Primary clinical outcome measures are assessed using the HRSD-24 and primary cognitive outcome measures are assessed using the Autobiographical Memory Test (AMT). The secondary objective is to evaluate the efficacy of MST compared to RUL-UB ECT in ameliorating suicidal ideation in patients with MDD. The secondary hypothesis is that MST will have a non-inferior remission rate on the SSI compared to RUL-UB-ECT in patients with depression. Secondary clinical outcome measures include the SSI. Time to reorientation, measuring patients’ reorientation time after treatment, will be completed after the first three treatments. For a detailed overview of all study assessments and information about the schedule of measures in relation to the study timeline, see Table 1. In addition, a tertiary objective for this trial involves the development of candidate neurophysiological biomarkers, which may predict response to treatment [36].

### Statistical methods

We are using a combined primary effectiveness endpoint where (1) MST will result in remission rates on the HRSD-24 that is non-inferior to that of RUL-UB ECT; AND (2) MST will have a superior cognitive adverse effect and tolerability profile compared to RUL-UB ECT assessed with the AMT.

In the primary outcome analysis, baseline variables will be summarized for each group by descriptive statistics. As per Senn et al. and Pocock et. al. [53, 54], significance tests between groups on baseline characteristics in a randomized trial are ill-advised and will not be done.

The primary efficacy analysis will be carried out in two stages. The first stage will test for non-inferiority of MST compared to ECT on remission. The second stage is a superiority comparison on cognitive function. If non-inferiority is established, then the second stage analysis will be carried out. This closed testing procedure ensures that the type I error will not exceed the nominal alpha of 0.05.

The stage 1 analysis hypothesis to be tested is H_0: π_ECT-π_MST≥0.15 versus H_1: π_ECT-π_MST< 0.15 where π_ECT and π_MST are the probabilities of remission in the ECT and MST groups respectively. The primary comparison will be a one-sided *Z*-test in difference of proportions, compared against the non-inferiority margin of 15%. The 15% non-inferiority margin was chosen as a remission rate of 35% remains clinically meaningful in this difficult to treat sample and is still higher than more conventional, less invasive treatments for treatment-resistant depression (e.g. rTMS at about 20% remission [55] and 14% with antidepressants [56]). The absolute risk difference will be calculated along with 90% and 95% confidence intervals, using standard normal approximations. Since ITT analysis introduces a conservatism that is undesirable for non-inferiority trials, both ITT (i.e. all randomized participants) and completer analyses (i.e. 8 treatments or met remission criteria) will be performed. The primary analysis for Stage 1 will be a completer analysis and a sensitivity ITT analysis will be done secondarily. We plan to collect the primary outcome data from all patients regardless of treatment compliance to minimize missing data in the outcomes. If outcomes are missing in more than 5% of the patients in the ITT analysis, inverse probability weighting will be employed to assess and mitigate the effect of missing data. It is only necessary for non-inferiority to be established in this analysis in order to proceed to the stage 2 analysis although additional secondary analyses of this outcome will be performed.

An adjusted analysis will be performed using generalized linear models for binary data. Clinical variables known to be associated with remission will be included (see again Senn et al. [53] and Pocock et. al. [54]). The following variables will be included in the analysis, which have been shown to be related to response/remission in this population: number of failed antidepressant trials, duration of most recent major depressive episode, number of major depressive episodes, and benzodiazepine use. The purpose of the adjusted analysis is to ensure the robustness of the non-inferiority findings when accounting for the variance in our model attributable to predictors of outcome. This will be examined in two ways. First, the unadjusted odds ratio and 95% confidence interval will be compared with the adjusted for consistency. Second, the fitted logistic regression model will be used to estimate the probability of response for each subject. An adjusted difference in proportions will be estimated by averaging the predicted probabilities within group and taking the difference. A bootstrap will then be used to generate a distribution of this measurement from which the bias corrected percentiles can be obtained to compare with the 15% non-inferiority margin.

Upon the determination of non-inferiority, we will move to stage 2 of the analysis which will examine cognitive superiority using the AMT. The binary outcome is defined as a worsening of > 25% on the AMT total score. The hypothesis to be tested is H_0: π_ECT-π_MST=0 versus H_1: π_ECT-π_MST≠0 where π_ECT and π_MST are the probabilities of deterioration in the ECT and MST groups respectively. This primary analysis will be ITT. The hypothesis will be tested with a chi-square test. The absolute risk difference and 95% confidence interval will be computed using standard methods.

Our secondary effectiveness endpoint outlines that MST will have a non-inferior remission rate on the SSI compared to RUL-UB-ECT in patients with depression. This will be determined using similar methods to those described above.

### Sample size

Our sample size calculations are based on non-inferiority trial calculations that are sufficiently large enough to minimize type II error [54] and are consistent with previous large, multi-centre ECT trials [44]. RUL-UB ECT and MST have been found to achieve remission in approximately 60% of patients with TRD [57, 58]. Non-inferiority trials, such as this study, specify a tolerance threshold, with a tolerance of 15% denoting equivalence between the two treatments when the effectiveness of MST can be concluded to be not less than 35%. The total sample size is derived as a function of tolerance and power with a significance level of 0.05. Using these methods, a total sample size of 260 participants (130 per group) yields 80% power to confirm a non-inferior difference in HRSD-24 remission rates of 15% between the two study groups. This sample size also provides > 95% power to detect a minimally important difference > 25% (absolute risk difference) change on the AMT total score. The primary analysis will proceed once a minimum of 260 participants achieve an adequate trial of treatment. The overall rate of dropout in a similar past trial at CAMH was less than 3%.

### Adverse event analysis

The analysis of all AEs will include incidence tables by severity, relationship to treatment and baseline parameters. AE rates will be compared between the study groups. We anticipate that there will be no significant differences in side effects between these two treatments and will conduct comparisons of safety endpoints at trial conclusion.

### Data management

Study staff are trained to collect complete and accurate source data and document all participant information in study-specific case report forms. Data is stored in an electronic data capture system, Medidata Rave (RAVE), designed specifically for the needs of our study. The study data manager and AHRC at the Li Ka Shing Knowledge Institute of SMH in Toronto, Canada, will manage the trial database. The electronic data capture system was designed to automatically complete range checks for all values and flag any deviant entries. The study data manager will conduct routine monitoring of study data including adherence to the protocol, data completion, and AE. All database activity is tracked through the electronic audit trail maintained by the database. Data quality checks of paper source documentation are conducted by verifying that the transcription into the eCRF database has been properly complete for two participants selected at random out of every 10 participants.

### Confidentiality

Participants are given a unique study ID upon entry and no identifying information is stored on study documents or within the RAVE database. Any personal identifying information is stored in a locked file on secure servers, at each respective site. Personal information is not shared between sites.

### Safety monitoring

Proactive site monitoring is overseen by the Office of Clinical Research (OCR) through the NIMH prior, during, and after the study to ensure that GCP is followed and maintained throughout the duration of the study. The OCR regularly visits both sites for trial oversight and is responsible for the creation and maintenance of a data safety monitoring board (DSMB). The DSMB is comprised of an independent group of researchers and experts based out of the NIMH. Its role is to monitor patient safety and treatment efficacy data during the conduct of this trial. Members of the group meet regularly, approximately every 4 months in order to review participant safety, study conduct, and study progress.

Throughout the study, notification of any serious adverse events (SAEs) and any proposed investigator-initiated changes in the protocol are submitted to the NIMH DSMB, the US FDA and Health Canada. The NIMH DSMB may at any time request additional information from the PI. All SAEs and AEs will be tabulated and submitted to the NIMH DSMB, and central and local research ethics boards in the triannual DSMB data reports or at the time of study continued review. Based on a review of safety data, the NIMH DSMB can issue directives concerning the conduct of the study.

Convulsive therapy is an involved treatment and there are potential side effects that are anticipated over the course of the trial. Safety and AEs are queried and documented at each study visit. The following AEs are anticipated in a sub-sample of the participant population: reversible cardiac ectopy, transient hypertension, uncomplicated asystole, fatigue, headache, aching/stiffness in muscles, nausea and vomiting, acute post-treatment delirium, post-ictal agitation, disorientation, neurocognitive impairment (e.g. anterograde and retrograde amnesia), prolonged seizures, treatment-emergent mania, treatment-emergent anxiety and fear, laryngospasm, peripheral nerve palsies, and aspiration, wakening paralysis, intravenous (IV) infiltration, other complications due to anaesthesia (e.g. sore throat, headache, shivering), dental injury, lip lacerations and falls. All AEs are recorded and reported to the site PI for consideration of further action. Participants receive care as appropriate for any harm that arises as a result of study participation.

### Dissemination plan

This study is conducted in accordance with the publication and data sharing policies and regulations of the National Institute of Health (NIH) Public Access Policy. This policy requires scientists to submit final peer-reviewed journal manuscripts that arise from NIH funds to the digital archive PubMed Central upon acceptance for publication. This study will also comply with the NIH Policy on the Dissemination of NIH-Funded Clinical Trial Information and US FDA Clinical Trials Registration and Results Information Submission rule. As such, this trial is registered at ClinicalTrials.gov.

Data from this study is submitted to the NIMH Data Archive (NDA) approximately every 6 months. The NDA is a data repository operated by the NIMH that allows researchers studying mental illness to collect and share deidentified information with each other. During and after the study, the researchers will send deidentified information collected from participants to NDA.

A further goal of this research is to inform and educate the wider community, both professional and public, about the potential of MST in the treatment of MDD/TRD. To this end, the study team will present the findings of this research at both national and international conferences and submit results for peer-reviewed publication to affirm the significance of potential findings. In addition, the study team will foster awareness in the community through the dissemination of any results in an accessible manner to help educate and promote understanding of convulsive therapy in general and MST in particular.

## Discussion

Patients with TRD in the USA use more health care resources and have significantly higher overall health care payments compared to non-TRD patients [59]. Yet treatment options for this population are limited; moreover, those available antidepressant therapies such as ECT, are highly stigmatized and produce significant cognitive adverse effects such as amnesia and executive dysfunction [6]. An alternative first-line convulsive therapy, such as MST, with high remission rates in severely ill patients and those with TRD would be transformative for the field. The specific aims of the protocol described in this paper are to conduct a randomized non-inferiority trial evaluating the efficacy, tolerability, and cognitive adverse effects of two different forms of convulsive therapy (MST and RUL-UB ECT) for MDD/TRD, to assess the efficacy of two forms of convulsive therapy in ameliorating suicidal ideation in patients with depression, and to identify a neurophysiological biomarker of a clinically meaningful treatment response indicator. If MST demonstrates comparable efficacy to ECT, but with cognitive safety, it should be rapidly adopted into clinical practice as providers, patients and their families may be far more likely to accept this treatment.

The strengths of our protocol include (1) the use of a new treatment option, MST, for difficult to treat depression complete with preliminary evidence that supports its safety and efficacy; (2) intensive clinical and neurocognitive study assessments pre-, during-, and post-treatment to monitor progression and ensure safety of participants; (3) longitudinal follow-up post-treatment to monitor long term outcomes of patients who did and did not respond to convulsive therapy; (4) trial oversight by an interdisciplinary team of experts in convulsive therapy across two leading North American institutions along with study monitoring by the NIMH OCR; and (5) a linked non-inferiority/superiority gated analysis statistical approach and comprehensive dissemination plan.

Although this protocol is clinically significant and strong, there are some limitations. First, recruitment is limited to two institutions in North America: CAMH and UT Southwestern. That being said, efforts to recruit a representative and generalizable study sample will be made by both study sites from the outset. Through our study design, careful data collection practices, and the appropriate statistical analyses, our data will be internally valid allowing us to draw firm conclusions about the true treatment effect in our population of interest. Second, historically it has been shown in multiple studies that the acceptability of convulsive therapy as a treatment option is underrepresented in minority populations as opposed to patients who identify as white [60, 61]. As outlined above, we will endeavour to recruit an ethnically diverse sample across both sites; however, this may prove challenging based on prior study findings. Our efforts in community engagement and education as described in our dissemination plan will go a long way towards fulfilling this aim and dispelling some of the stigma associated with convulsive therapy.

Recently published evidence from our open-label pilot clinical trial conducted at CAMH provides promising support for MST as an effective and safe treatment for adult patients with difficult to treat depression. For example, patients who received high-frequency MST (100 Hz) achieved response and remission rates of 41.7% and 33.3%, respectively, for adequate trial completers (i.e. 8 treatments or more); and 60% and 53.3%, respectively, for protocol completers (i.e. 24 treatments) [35]. For both groups, the remission rates with high-frequency MST were significantly greater than the remission rates for low-frequency (25 Hz) MST supporting further investigation of 100 Hz MST. Additionally, an important use of ECT is rapid relief of acute suicidal ideation, and Weissman et al. (2020) found remission of suicidal ideation was achieved in 47.8% of patients who endorsed suicidality at baseline in the MST open-label trial[62]. Findings from the pilot study also suggested that MST is cognitively safe [35]. Thus, should the clinical efficacy and safer cognitive effects of MST be replicated in this study, our results could lead to confirmation of a new potential treatment alternative to ECT.

The underlying interest in MST is that it is expected to have better cognitive outcomes than ECT and so would be preferred if it is at least as effective in relieving clinical symptoms as ECT is. This reasoning implies that unless MST is no worse than ECT with respect to clinical symptoms, its impact on cognitive outcomes is not important given MST would not be indicated if it is worse than ECT. The staged approach to the analysis reflects these realities and permits both questions to be answered in a single trial rather than two trials; a non-inferiority trial for clinical outcomes follow by a superiority trial for cognitive outcomes. With the staged approach, a sufficient sample size, and valid data, our statistical analyses will be robust and answer the underlying clinical management question. If our hypotheses are confirmed, the results of this trial will be presented to the FDA in support of marketing MST for patients with TRD.

Our study team has put forth great effort to ensure data collected from the two sites will be generalizable and accurately reflect the treatment effect and clinical practice for the US population. Furthermore, our inclusion and exclusion criteria have been designed to closely mirror the typical clinical presentation of the population that receives ECT and that has been used in previous ECT treatment trials [41, 44, 45, 63]. This strategy will help to maximize both the internal and external validity (i.e. generalizability) of our findings. This study has been accepted by the US FDA in support of a 510(k) premarket notification of intent to market the MagVenture MST MagPro XP with Cool Twin Coil for patients with depression. The MagProXP has already received International Electrotechnical Commission certification and we expect a marketing submission for this device to be submitted to the US FDA approximately 6 months after study completion. Data generated from this study will be pivotal in supporting the approval of MST for use in TRD and accessibility of this potentially ground-breaking new antidepressant treatment to a wider population.

In conclusion, the CREST-MST protocol was predicated on existing pilot data that supported the safety and efficacy of MST for MDD and through strong collaborations with an integrated healthcare study team including experts in the fields of psychiatry, convulsive therapy, TRD, anesthesiology, clinical neuropsychology, and neurophysiology. Should the results of this trial support MST as non-inferior and cognitively superior to ECT, MST could become a standard antidepressant treatment for patients with TRD. Existing ECT suites can easily accommodate MST without major modifications. Indeed, given that the administration of MST is nearly identical to ECT, the majority of ECT facilities in North America would be able to readily adopt MST into their established convulsive therapy programmes. By establishing MST as a safe and effective treatment for severe depression, this trial will provide a new antidepressant therapy to advance the care of depression in adults and improve overall health outcomes.

## Supplementary Information

**Additional file 1:.** SPIRIT checklist

**Additional file 2:.** CREST-MST Consent

## Data Availability

The final de-dentified dataset generated from the current protocol will be available as part of the NIMH Data Archive (NDA) and from the corresponding author on reasonable request.

## Abbreviations

AE: Adverse event
AHRC: Applied Health Research Centre
AMT: Autobiographical Memory Test
CAMH: Centre for Addiction and Mental Health
CONSORT: Consolidated Standards of Reporting Trials
DSMB: Data safety monitoring board
ECT: Electroconvulsive therapy
ECS: Electroconvulsive seizure
EEG: Electroencephalography
FDA: Food and Drug Administration
FDG-PET: Fluorodeoxyglucose positron emission tomography
GCP: Good Clinical Practice
HRSD-24: 24-item Hamilton Rating Scale for Depression
IDE: Investigational Device Exemption
ITA: Investigational Testing Authorization
ITT: Intention to treat
IV: Intravenous
MDD: Major depressive disorder
MINI: Mini-International Neuropsychiatric Interview
MS: Magnetic seizure
MST: Magnetic seizure therapy
NIH: National Institute of Health
NIMH: National Institute of Mental Health
NDA: NIMH Data Archive
OCR: Office of Clinical Research
PI: Principal Investigator
RAVE: Medidata Rave
RUL: Right unilateral
RUL-UB-ECT: Right unilateral ultrabrief ECT
rTMS: Repetitive transcranial magnetic stimulation
SAE: Serious adverse event
SMH: St. Michael’s Hospital
SSI: Beck Scale for Suicidal Ideation
SPIRIT: Standard Protocol Items: Recommendations for Interventional Trials
TRD: Treatment-resistant depression
UT Southwestern: University of Texas Southwestern
