# Supplementary material for: Confirmatory Efficacy and Safety Trial of Magnetic Seizure Therapy for Depression (CREST-MST): study protocol for a randomized non-inferiority trial of magnetic seizure therapy versus electroconvulsive therapy
Source: Trials. 2021 Nov 8;22:786. doi: 10.1186/s13063-021-05730-7 (PMC8576983; doi:10.1186/s13063-021-05730-7)
Supplement: Supplementary file 2 — Additional file 2:. CREST-MST Consent [file 13063_2021_5730_MOESM2_ESM.pdf]

INFORMED CONSENT AND INFORMATION SHEET

Confirmatory Efficacy and Safety Trial of Magnetic Seizure Therapy for Depression: CREST-MST

Dr. Daniel Blumberger

This Informed Consent form has 2 parts:

- Information Sheet (to share information about the research with you)
- Certificate of Consent (for signatures if you agree to take part)

You will be offered a copy of the full informed consent form.

Part I: Information Sheet

Introduction

You are being asked to take part in a research study. Please read this explanation about the study and its risks and benefits before you decide if you would like to take part. You should take as much time as you need to make your decision. You should ask the study doctor or study staff to explain anything that you do not understand and make sure that all of your questions have been answered before signing this consent form. Before you make your decision, feel free to talk about this study with anyone you wish. Participation in this study is voluntary. There is a section at the end of this document that explains how the study may be conducted remotely when extra precautions are required and how some of the procedures may differ.

Background and Purpose

- You have been asked to take part in this research study because you have a diagnosis of Major Depressive Disorder and are interested in pursuing brain stimulation therapy, as prescribed by your clinical physician.
- Electroconvulsive Therapy (ECT) is the most established type of brain stimulation therapy, and uses electrical stimulation to stimulate the brain and induce a seizure.
- ECT is effective to treat depression but may cause memory loss.
- With this study we would like to investigate a recently developed treatment, Magnetic Seizure Therapy (MST). It is hoped that MST will prove to be as good as ECT in treating depression, but with less memory loss.
- For both treatments, a seizure is induced while you are under general anesthesia.

- At this time, MST is an investigational treatment and has not yet been approved by Health Canada for clinical use outside of research studies like this one. ECT, on the other hand, has been in use for several decades and there is extensive research documenting its efficacy.
- This study will compare these two different treatments to see if they have the same or different effectiveness in treating major depression. If MST proves to be as good as ECT in treating depression, but with fewer side effects including memory loss, it will be an important treatment option in future years.
- About 260 people will participate in the study. They will come from the University of Texas – Southwestern Medical Center and the Centre for Addiction and Mental Health.
- If new information emerges about the treatments in this study that alters the risks or benefits of participation you will be notified.

Study Design

- This study compares the effectiveness of two different kinds of treatments: MST and ECT.
- Whether you receive MST or ECT will be decided randomly (by chance). The number of people getting treatment in each study group will be ~130, so you will have a 1 in 2 chance of receiving MST and a 1 in 2 chance of receiving ECT.
- This study will be blinded. This means that neither you nor the study team member, who follows your progress, will know which kind of treatment you are receiving until the study is finished. This information can be found out at any time in case of an emergency.
- You will undergo treatment in this study for approximately 7 weeks (or a maximum of 21 treatments).
- There will be one screening visit lasting around 1 – 1.5 hours, and two baseline visits, each lasting between 1 - 1.5 hours.
- There will be a maximum of 21 treatment visits (Monday, Wednesday and Friday for 7 weeks). There will be a post-treatment visit conducted after you end treatment, lasting approximately 3 hours. There will also be a follow-up visit 6 months after treatment completion (lasting approximately 3 hours).
- You will also have the choice to complete an additional component involving testing sessions before and after your treatment course that examine your brain's physiology. These processes are known as cortical inhibition and multi-scale entropy. These sessions will only be offered if non-essential research is deemed safe to conduct, according to hospital directives.

Important Precautions

You should be aware that the magnetic fields generated by the stimulator might damage magnetic cards, watches and some electrical devices. *Please remove any such items before testing*.

Exposure to magnetic stimulation or any strong magnetic field is **not permitted** in people who have a pacemaker, an implanted medication pump, a metal plate in the skull, or metal objects inside the eye or skull (for example, after brain surgery or a shrapnel wound). *Please inform investigators if you have any of these*.

Study Visits and Procedures

Screening Visit: The first study visit will be a screening visit. This visit will involve an interview to confirm your diagnosis of major depressive disorder and rule out other psychiatric diagnoses that might interfere with treatment, and to confirm that you can safely undergo MST or ECT. The results of the tests/questions at the screening visit help the researchers to decide whether you can continue in this study. The results of all tests and interviews are completely confidential. There are also clinical procedures which you will be asked to complete, including bloodwork and an electrocardiogram (ECG). Additionally, a consult with the anesthesiologist will be scheduled to review your bloodwork and ECG, and ensure that it is medically safe for you to undergo anesthesia.

Baseline Visit: This visit will be conducted after your screening visit once your eligibility for this study has been confirmed. You will undergo some interviews and fill out questionnaires regarding your symptoms. You will also complete about one and half hours of cognitive assessments that will assess your memory and executive functioning. These require two separate visits both located here at the Queen St. location of CAMH.

Treatment Visits 1-21: The treatments will take place over a 7 week time frame, three days a week (Monday, Wednesday and Friday). You will be asked to attend for treatment in the morning when the clinic opens. The treatment procedure is approximately 10 minutes, followed by a recovery period of approximately 30 minutes until you are stable and feeling well enough to be escorted home. Please note that the duration of your visit to CAMH will vary based on wait times in the clinic. *It is required that you have an escort to take you home after each treatment visit. An escort may be a relative, friend, neighbour, case worker, etc. A taxi driver is not considered a suitable escort.*

If you miss more than 2 scheduled treatment sessions, your treatment as part of the study will be stopped, as missing treatment sessions compromises the efficacy of the treatment.

Monitoring Visits: The study team will follow your progress with additional monitoring visits after every 3 - 4 treatments. It should take around 30-45 minutes to complete this monitoring visit.

Post-Treatment Monitoring Visit: After your final treatment you will undergo the same interviews and questionnaires as you did in your baseline visit, as well as the cognitive tasks to measure your executive function. This visit should take around 2.5 – 3 hours.

Follow-up Monitoring Visits: A final follow-up visit, lasting around 3 hours, will take place 6

months after you end treatment.

Should you withdraw or be discontinued from treatment prior to completing the full course, we aim to continue to follow you at the regularly scheduled time points described above. This allows us to collect more complete data regarding the efficacy of these treatments.

Additional Sessions to Measure Brain Physiology: Transcranial magnetic stimulation (TMS) is a method used to measure brain inhibition. TMS excites nerves over the area of the brain involved in moving your hand muscles. When the nerves are stimulated, this causes the muscles in your hand to move, which will be recorded and later analyzed. Brain activity and inhibition during TMS will be assessed using electroencephalography (EEG) and electromyography (EMG).

- You will be seated in a comfortable chair and we will attach soft foam electrodes to the skin surface over your hand muscles; these electrodes will then be connected to a recorder that will record the activity of your hand muscles.
- It takes approximately 30 minutes to put on the EEG cap and get it ready for recording. The cap contains many recorders that record your brain activity. There is gel on the inside of the cap that may be sticky; you will be allowed to rinse it out after the test.
- A magnetic coil will be held on the surface of your scalp
- When the magnetic stimulation is applied, you will feel a twitch or small movement in your hand, but there should be no pain.
- The TMS measures of brain physiology will be taken from your motor cortex (the part of the brain that controls movement) and the prefrontal cortex (the part of the brain that controls thinking).

If you choose to participate in these sessions, there will be one scheduled before your first treatment session, and one after your treatment course is complete, each lasting around 2 – 2.5 hours. As mentioned above, these sessions are optional and will only be offered if non-essential research is deemed safe according to hospital directives.

Calendar of Visits

Boxes marked with an X show what will happen at each visit:

**Visit**	Interview and Question-naires	Cognitive Assessments	Clinical Lab[e]/ Consult	TMS-EEG (optional)[f]	Treatment (MST or ECT)	Time
Screening Visit	X		X[d]			1.5 – 2.5 hours
Baseline Visit 1	X		X			1 hour

Baseline Visit 2		X		X (2 – 2.5 hours)		1.5 hours
Visits1-21[a]					X	
Visits 4, 7, 10, 13, 16 and 19[c]	X				X	30-45 minutes[b]
Post-treatment Monitoring Visit	X	X		X (2 – 2.5 hours)		2.5-3 hours
6 month post-treatment Monitoring Visit	X	X				2.5-3 hours

[a]For these visits, we cannot provide an exact estimate of the total duration of time, as this will be affected by wait times at the facility on a given day.
[b]The time duration listed for visits 4, 7, 10, 13, 16 and 19 only accounts for the interview and questionnaire portion.
[c]There is some flexibility with the interview and questionnaire monitoring visits, as they may be rescheduled to facilitate treatment scheduling.
[d]This will include an anesthesia consult, which will likely require that you are on site for longer.
[e]Clinical labs may be completed at a time point other than the Screening Visit, but this information should be available for review at the first visit.
[f] These sessions are optional and will only be offered if non-essential research is deemed safe according to hospital directives.

Planning for Treatment Visits

Because you will be receiving a general anaesthetic you cannot have anything to eat or drink after midnight the night before your treatment.

You MUST have an escort to take you home after each of your treatments. An escort may be a relative, friend, neighbour, case worker, etc. A taxi driver is not considered a suitable escort.

Prior to treatment start you will be seen by a brain stimulation physician, during this visit you may be asked to change a medication that could interfere with the MST or ECT procedure. The limiting or discontinuation of any medications prior to treatment start is a clinical decision and all risks associated with this will be reviewed with the brain stimulation physician or your clinical physician.

Taking a benzodiazepine at a dose greater than lorazepam 2 mg or equivalent or taking a non-benzodiazepine anticonvulsant medication is not permitted in the study as it could interfere with the MST or ECT procedure

Should you be required clinically to start any new medication, please ensure to inform the research team. We also ask you to please refrain from starting any new “over the counter” or “as needed” medications without discussing this with the research team first. This includes medication to treat anxiety or insomnia such as lorazepam or clonazepam.

After each treatment, you will spend some time in the recovery room where your vitals will be measured, nursing staff will monitor your status and you will be asked some questions by study staff to check your orientation (e.g. name, DOB, place, etc.). There will be other patients in this space who will be at various stages of recovery and reorientation. Current practise standards aimed at maintaining confidentiality will be applied throughout this process.

Reminders

It is important to remember the following things during this study:

- Ask your study team about anything that worries you.
- Tell study staff anything about your health that has changed.
- Tell study staff if you are considering any changes to your medications or doses.
- Tell study staff if you have changed any of your medications or doses.
- Tell study staff if you become pregnant during the study.
- Tell study staff if your depression becomes worse.
- Tell study staff if you are having thoughts about hurting yourself or anyone else.
- Tell study staff if you have noticed changes to your memory
- Tell your study team if you change your mind about being in this study.

Risks Related to Being in the Study

Based on safety studies over the last decade, the known risks of the magnetically-induced seizures of MST are not greater than the known risks of electrically-induced seizures in ECT (these are summarized below). There is a possibility that you may also experience a worsening of your symptoms during the course of the trial. You may experience side-effects after each treatment. This can be caused by the treatment itself, the anesthetic medication or not having anything to eat or drink for a long period of time. The most common side-effects are headache, dizziness, nausea or vomiting, muscle aches and fatigue. There is also a small risk of experiencing difficulty breathing after each treatment. If this happens, the treating physician and anesthesiologist will adjust the doses of the medications administered during the treatment to ensure that this does not happen again. Some of these side effects can be reduced, therefore we encourage you to let the research staff know if you experience any of these. Treatment is available and will be provided by medical staff in the event of study-related injury or adverse event.

Known risks of electrically-induced convulsive therapy include:
1) Risks of general anesthesia, which involves about 1/100, 000 treatments risk of death.
2) Risks of non-terminating seizures in about 1/1000. This is usually treated with a medication

given to the patient by the doctor monitoring the anesthesia.
3) Risks of decreased heart rate in about 1/1000. This usually resolves on its own.
4) Risks of high blood pressure in about 1/1000. This usually resolves on its own but is monitored. The anesthetist may also administer a fast-acting medication to reverse severe high blood pressure.
5) Risks of increased brain pressure in about 1/100, 000. This effect, again, is usually temporary, and patients with conditions that would increase this risk are identified during the initial ECT screening.
6) Risks of temporary decline in blood oxygen during the seizure. Again, this is usually temporary and is monitored throughout the duration of the seizure.
7) Risks associated with a confusional state, including agitation and risk of injury due to falls from the ECT bed following the seizure. This is minimized by close supervision. In rare cases, brief sedation is used to avoid fall/injury.
8) Risks of emergence of hypomania or mania. A measure of manic symptoms is included in the monitoring visits to screen for this.
9) ECT and MST can cause confusion and memory loss. This may start immediately after treatment and may continue after treatment is stopped. Memory loss can affect new memories (for example, it may be hard to learn or remember new information) or old memories (you might not be able to remember past experiences or other memories). These side effects can range from mild (unsettling to the participant) to severe (impact daily activity and cause distress for participants).

You may find it helpful to write down things you want to remember and use a calendar for appointments and important details. You may want to keep important contact information in a place you can easily find. The study team will also provide you with a wallet card detailing study contact information. Please talk to the study team if you have any questions or are worried about this.

The overall mortality rate for ECT is currently around 1 per 100, 000 treatments according to statistics maintained by the American Psychiatric Association.

If you have further concerns, you may contact your study doctor at any time.

A study physician will be present at all times during the treatment component of this study and will promptly assess and treat any side effects you may experience in consultation with one of the anesthesiologists.

Incidental Findings

Research scans are not subject to clinical review and the psychological test and interviews are not used for diagnostic purposes. However, any incidental findings will be communicated to you and, upon your request, to your clinical physician.

Risks Related to Pregnancy

There are no known risks of MST or ECT during pregnancy. However, there is always a possibility that if you are pregnant, MST or ECT may have risks that we do not know about. For this reason, you cannot participate in the study if you are pregnant. We also ask that you not actively try to become pregnant while participating in this trial.

Benefits to Being in the Study

You may or may not receive any direct benefit from being in this study. MST or ECT may improve your symptoms of depression, or may have no effect. Information learned from this study may help other people undergoing a convulsive therapy for major depression in the future.

Right to refuse or withdraw from the study

Your participation in this study is voluntary. You may refuse to participate or stop your participation at any time without penalty and without jeopardizing your continuing medical care at this institution.

Should you choose to withdraw from the treatment portion of the study or are unable to finish all of the scheduled treatment appointments due to unforeseen circumstances, you may still agree to participate in the remaining clinical assessments as scheduled.

Throughout your participation in this study you may continue your regular appointments with your original treating physician.

Discontinuation

It should be noted that your treatment may be discontinued without your consent under the following conditions:

- if you miss more than two scheduled treatment sessions
- the study physician decides to stop your convulsive therapy treatment for safety reasons
- you are lost to follow-up
- you are not compliant with the requirements of the study, which include the inclusion/exclusion criteria
- the study is stopped or halted prematurely
- the investigator believes that it is in your best interest (e.g., you experience a serious adverse event) to stop treatment
- you become pregnant
- you experience a significant worsening in depression
- you experience a significant increase in suicidal ideation with imminent intent or attempt suicide
- you develop signs of mania or hypomania

- a seizure cannot be induced on two consecutive treatment sessions, despite optimization of treatment parameters and anesthesia

If you stop treatment early, we will complete the scheduled post-treatment and follow-up visits as outlined above.

Alternatives to Being in the Study

You do not have to join this study to receive treatment for your condition. ECT is offered in a non-research setting at various hospitals in Toronto and elsewhere. There are also many other approved medications/interventions for major depression:

- There are many antidepressant medications that are available alternatives to participation in this study. Your psychiatrist or family doctor can help you decide on the best antidepressant medication for your illness.
- Psychotherapies such as cognitive behavioral therapy (CBT), interpersonal therapy (IPT), or mindfulness-based cognitive therapy (MBCT).
- Other forms of brain stimulation therapy such as bilateral electroconvulsive therapy, for depression that is severe or unresponsive to other treatments.
- There are also other research studies looking at other brain stimulation treatments for your condition.
- You may also choose not to have any treatment for your condition.

Your doctor will discuss any of these options with you.

Confidentiality

The confidentiality of the data collected and identity of the individuals participating in this study will be strictly maintained. The names and identity of the participants will not be revealed in any discussion of this work.

To determine if you meet the requirements to participate in this study, a member of the research team will need to access your CAMH Health Record.

As part of continuing review of the research, your study records may be assessed on behalf of the Research Ethics Board. A person from the research ethics team may contact you (if your contact information is available) to ask you questions about the research study and your consent to participate. The person assessing your file or contacting you must maintain your confidentiality to the extent permitted by law. The information regarding this clinical trial will be entered into a databank.

As part of the Research Services Quality Assurance Program, this study may be monitored and/or audited by a member of the Quality Assurance Team. Your research records and CAMH records may be reviewed during which confidentiality will be maintained as per CAMH policies and to the extent permitted by law.

A description of the research study will be available at www.clinicaltrials.gov. This website will not include information that can identify you in any way. At most, the website will include a summary of the results. You can search this website for the study at any time.

Data from this study may be submitted to the National Institute of Mental Health Data Archive (NDA). NDA is a data repository run by the National Institute of Mental Health (NIMH) that allows researchers studying mental illness to collect and share deidentified information with each other. A data repository is a large database where information from many studies is stored and managed. Deidentified information means that all personal information about research participants such as name, address, and phone number is removed and replaced with a code number. With an easier way to share, researchers hope to learn new and important things about mental illnesses more quickly than before.

During and after the study, the researchers will send deidentified information about your health and behavior and in some cases, your genetic information, to NDA. Other researchers nationwide can then file an application with the NIMH to obtain access to your deidentified study data for research purposes. Experts at the NIMH who know how to protect health and science information will look at every request carefully to minimize risks to your privacy.

You may not benefit directly from allowing your information to be shared with NDA. The information provided to NDA may help researchers around the world treat future children and adults with mental illnesses so that they have better outcomes. NIMH will also report to Congress and on its web site about the different studies that researchers are conducting using NDA data. However, you will not be contacted directly about the data you contributed to NDA.

You may decide now or later that you do not want to share your information using NDA. If so, contact the researchers who conducted this study, and they will tell NDA, which can stop sharing the research information. However, NDA cannot take back information that was shared before you changed your mind. If you would like more information about NDA, this is available on-line at http://data-archive.nimh.gov.

Personal Health Information

If you agree to join this study, the study doctor will look at your personal health information for clinical reasons only. Your personal health information will not be recorded in the study records. Personal health information is any information that could be used to identify you and includes your:

- name,
- address,
- date of birth,
- new or existing medical records, that includes types, dates and results of medical tests or procedures.

The information that is collected for the study will be kept in a locked and secure area (secure

server for electronic data) by the study doctor for 25 years. Only the study team or the people or groups listed below will be allowed to look at your records. Your participation in this study will also be recorded in your medical record at this hospital. The following people may also come to the hospital to look at the study records and at your personal health information to check that the information collected for the study is correct and to make sure the study followed proper laws and guidelines:

- Representatives of Canadian, U.S. or health regulatory agencies (e.g. Health Canada, Food and Drug Administration)
- Representatives of CAMH, UT Southwestern Medical Center or the National Institutes of Mental Health (NIMH)

All information collected during this study, including your personal health information, will be kept confidential and will not be shared with anyone outside the study unless required by law. If you consent to be re-contacted for future research studies, some basic information as described below may be shared with other authorized research personnel affiliated with the Temerty Centre. You will not be named in any reports, publications, or presentations that may come from this study. If you decide to leave the study, the information about you that was collected before you left the study will still be used. No new information will be collected without your permission.

With your permission, some basic information (e.g. demographics, medications, safety screening, brain stimulation history, etc.) gathered as part of the screening process will be stored in a centralized electronic database and may be shared with other research personnel affiliated with the Temerty Centre. This data will be used to safely track your participation and to better match you with current and future studies that you may be eligible for if you consent to be re-contacted. Only investigators/research teams affiliated with the Temerty Centre will have access to this secured database, and will adhere to all appropriate measures to safeguard the confidentiality of your information.

Because this is a treatment study, your signed consent form will be scanned and sent to the CAMH medical records department for your file.

New Information

If new information becomes available that is relevant to your participation to continue in the study, you will be informed in a timely manner.

In Case You Are Harmed in the Study

If you become ill, injured or harmed as a result of taking part in this study, you will receive care. The reasonable costs of such care will be covered for any injury, illness or harm that is directly a result of being in this study. In no way does signing this consent form waive your legal rights nor does it relieve the investigators, sponsors or involved institutions from their legal and professional responsibilities. You do not give up any of your legal rights by signing this consent

form.

Expenses Associated with Participating in the Study

You will not have to pay for any of the treatments or other procedures involved with this study.

Modified Procedures

All study visits, except for ECT/MST treatments and TMS-EEG sessions, will be conducted virtually using videocoference software (Webex), or the telephone if you are not able to use the videoconference software. Like online shopping, videoconferencing technology has some privacy and security risks. It is possible that information could be intercepted by unauthorized people (hacked) or otherwise shared by accident. This risk can't be completely eliminated, however CAMH has approved the use of WebEx for videoconferencing sessions because the appointments take place over a secure encrypted network. We want to make sure you are aware of this. The research team will confirm your identity at the beginning of the call and may also ask to see a piece of government-issued ID, via video, during the session.

Video sessions can be conducted using your cell phone, tablet or personal computer enabled with a camera/microphone and internet connection. You should use your home computer or personal device, and not a shared or work device, and use a home (private) Wi-Fi network, and not free (public) Wi-Fi for your internet connection. To use WebEx, an e-mail will be sent to you including the instructions for how to log-in. Self-report questionnaires will be completed using screen share, or verbally completed over Webex or telephone.

Do we have your consent to send you information by e-mail? The security of information sent by e-mail cannot be guaranteed.

☐ No

☐ Yes

The security of information sent by e-mail cannot be guaranteed. Please do not communicate personal sensitive information by e-mail. E-mail is not routinely monitored outside of work hours. Please do not use e-mail to communicate emergency or urgent health matters – please contact your clinician or family doctor. If it is a medical emergency, call 911.

For your safety, the research team will ask you for an emergency contact number, alternate phone number and your address before they start the call. They may follow-up with you after the session if you leave early. If at any time, we are concerned for your safety, we may contact you, your emergency contact or emergency responders to follow-up.
For these videoconference sessions, please try to find a quiet place where you can be by yourself and will not be disturbed and use earphones if you can. It's a good idea to test out the system a few minutes before the session to make sure the connection and sound are working. You or the research team can stop the session at any time, including if there are technical difficulties. If there are technical issues, one of our technical staff may join the call to provide

support. Some of the cognitive assessments cannot be completed remotely so the baseline, post-treatment, and follow-up visits will be shorter when they are conducted by video or telephone. During one of the cognitive assessments the research staff will take a screen shot of work you have done, your face will not be included.

ECT/MST treatments will be conducted in person. You will be told ahead of time what time you should arrive for your treatment in order to minimize the number of people in the waiting room. Prior to entering CAMH each day, every person is screened for COVID-19 symptoms and contacts. Any person who is found to have symptoms or contacts as per the screening protocol (known as a positive screen) will not be permitted to enter CAMH. If you are found to be a positive screen on any given day during the study, treatment will not be allowed to continue that day. Depending on the determination of the Infection Prevention and Control team at CAMH, we may or may not be able to resume treatments. If you miss more than 2 scheduled treatments due to a positive screen it will be at the discretion of the treatment team whether or not you can continue to receive treatment. If you do not have COVID-19 symptoms or contacts as per the screening protocol (known as a negative screen), you will be given a wristband to indicate you have been screened for that day. A CAMH staff will then escort you to the waiting room. Once you have recovered from the treatment the CAMH staff will take you to the person who is escorting you home.

During the optional brain physiology sessions, you will be required to wear a mask. The research staff will be wearing a mask, face shield and gloves. The research staff will maintain a physical distance whenever possible but there will be extended periods where they will be within 6ft/2m. All of the equipment and furniture that they use will be disinfected before and after the session. You will not be allowed to rinse out your hair at the end of the session, but we will provide you with a cap for your commute home.

Part II: Certificate of Consent

I have been invited to participate in this research treatment study and have read the attached information sheet. The discomforts and possible risks have been described to me. I understand that I can ask further questions during any stage of the study.

I understand that I may withdraw from the study at any time without affecting my treatment with my original treating physician. My identity will not be disclosed in any reference to the study or its results. I have also been offered a copy of the consent form.

Questions About the Study

Dr. Blumberger is responsible for the study. If you have any questions, please contact him at 416-535-8501 x33662. Dr. Robert Levitan (Chair, Research Ethics Board) is the external contact should you have further questions about participant rights. He can be reached at 416-535-8501 x 34020.

I voluntarily consent to participate in this study.

______________________________	____________________	____________
Participant's Name<br>(Print)	Signature	Date

______________________________	____________________	____________
Name of Person Obtaining Consent<br>(Print)	Signature	Date

I voluntarily consent to participate in two additional TMS-EEG sessions to measure cortical inhibition (*check here*) ☐
I do not agree to complete the optional TMS-EEG sessions (*check here*) ☐

______________________________	____________________
Signature	Date

I authorize ____________________ to disclose my personal health information,
(name of treating physician)
consisting of psychiatric or medical conditions, illnesses or psychiatric procedures that may influence my ability to participate in this study to the research analyst and/or Dr. Blumberger. I understand the purpose for disclosing this personal health information.

_____________________________ _____________________
Signature Date

Should I be interested in future research studies, I agree to be contacted in the future.

_____________________________ _____________________
Signature Date

Note- In addition, to ensure your safe participation in this study, you are being asked to designate someone whom the research team can contact about your depression symptoms and participation in the study, and who the research team can contact if they have questions about/are concerned about your well-being and/or are unable to reach you. This should be someone who has frequent contact with you and can contact the study staff in case of emergencies.

____
Name of contact person

___________________________ ________________________________
Telephone number Alternate telephone number

Or

□ I do not wish to provide a contact.
